# Gaze Estimation Method Using Analysis of Electrooculogram Signals and Kinect Sensor

**DOI:** 10.1155/2017/2074752

**Published:** 2017-08-20

**Authors:** Keiko Sakurai, Mingmin Yan, Koichi Tanno, Hiroki Tamura

**Affiliations:** ^1^Interdisciplinary Graduate School of Agriculture and Engineering, University of Miyazaki, Miyazaki, Japan; ^2^Faculty of Engineering, University of Miyazaki, Miyazaki, Japan

## Abstract

A gaze estimation system is one of the communication methods for severely disabled people who cannot perform gestures and speech. We previously developed an eye tracking method using a compact and light electrooculogram (EOG) signal, but its accuracy is not very high. In the present study, we conducted experiments to investigate the EOG component strongly correlated with the change of eye movements. The experiments in this study are of two types: experiments to see objects only by eye movements and experiments to see objects by face and eye movements. The experimental results show the possibility of an eye tracking method using EOG signals and a Kinect sensor.

## 1. Introduction

Gaze estimation has been an active research field for the past few years [1–3], and it is an important technique for severely handicapped people who cannot move the body or use speech to communicate [4, 5]. Some studies [6–11] have been developing various eye gaze interfaces using different eye movement recording methods. Examples are infrared oculography (IROG) [6–8], limbus tracking, and video oculography (VOG) [9–11].

We had proposed an eye tracking method using an electrooculogram (EOG) signal, which measures the potential across the cornea and retina [12–14]. With the EOG, the eyeball can be modeled as a dipole [15]. EOGs are widely applied in the medical field because they place a low burden on patients. The literature includes several EOG-based human-computer interface [12–14, 16, 17]. To investigate the possibility of using the EOG for a human-computer interface, the relation between the gaze angle and the EOG must be determined. However, in-depth studies [18, 19] have shown that the slowly changing baseline drift poses a difficulty for estimating continuous EOG signals, and this drift only appears in direct current (DC) signals in the circuit. We previously developed an EOG system using the center parameter update technique, which reduces baseline drift by segmentation of the signal [12]. The system that we developed [12–14] can possibly improve the communication abilities of patients who are able to move their neck and/or eyes; however, the low resolution of our system is a problem.

In the conventional method, the positions of the electrodes are set as plus channels in the same direction of the eye movements (e.g., [20, 21]). The horizontal channel records horizontal EOG signals and the vertical channel records vertical EOG signals.  Table 1 shows the electrodes positions of our method and the conventional method.

We have already proposed a cross-channel method to improve the accuracy of the EOG signal [14]. The method we already proposed [14] can classify four patterns based on alternating current (AC) and direct current signals and place the electrodes at locations away from the eyeball (Table 1). Although this method [14] is superior to the conventional method, paper [14] is pattern classification (up, bottom, right, and left) by a simple threshold method, and the direction of the face was not taken into consideration.

We previously developed an eye input application for a desktop PC with highly accurate gaze estimation [12]. Furthermore, we carried out large-space experiments (range: −60 degrees to 60 degrees) and estimated the gaze by multiple regression analysis using the DC integral value [22]. Although the regression analysis results were good, a narrower range would be preferable.

In the present study, we analyzed DC, AC, DC difference value, and DC integral value for regression analyses and we also checked whether the accuracy was improved over that in our previous studies [22]. Moreover, we considered the limiting angle of gaze estimation in a wide space ranging from −90 degrees to 90 degrees. Experiments were carried out under the condition that subjects can use only eyeball movements without moving the face and under the condition that the face and eyeballs can move freely.

In the experiment of the face moving freely, the position of the face was measured by two depth sensors and a RGB-D camera of the Kinect sensor [23, 24].

## 2. Measurement System

### 2.1. Using EOG Signals

**Figure 1:**
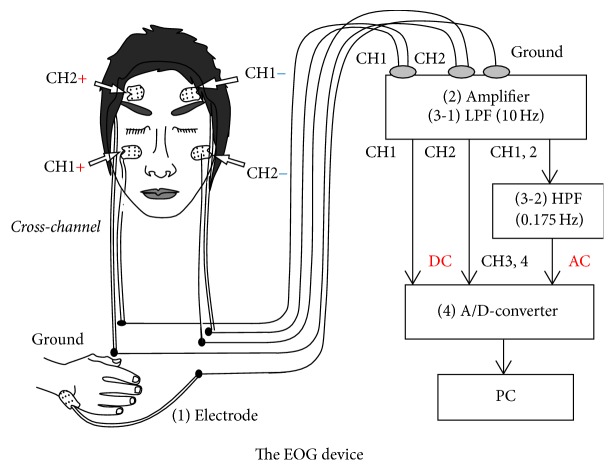
EOG measurement system.

In this section, the cross-channel EOG measurement system design [12] is shown.  Figure 1 shows the formal scheme for the acquisition and analysis of the EOG signal for the control and flow of information through the system. Our proposed system is based on the following six features: (1) five electrodes, (2) amplifier, (3) low-pass filter for channels 1 and 2, (4) high-pass filter for channels 3 and 4, (5) A/D-converter, and (6) PC and monitor. In order to effectively filter functions, channel 3 and channel 4 each use two amplifiers. The horizontal signals and the vertical signals can be recorded by both channels at the same time. This is an advantage because it is much easier to analyze data by using double simultaneous signals.

Baseline drift is the slow change of the EOG signals, in which the potential difference varies even if the eyeball position is constant. This drift only appears in DC signals and affects the EOG signal only slightly during fast eye movements (saccade). However, all other movements, such as fixations (when the eyes are still) and pursuit (when following a moving target), are affected by baseline drift.

Since the amount of change in the gaze direction directly corresponds to the amount of change in the DC signal of the EOG, the DC amplifier is generally used for EOG measurement. Therefore, the drift in the DC component is a big problem.

#### 2.1.1. The Differences between This Proposal Method and Paper [14]

The EOG system used in this paper is the same device as the EOG-EMG system of paper [14], but the following two points are different.

1. Paper [14] and the proposed method differ in EOG data handled and identification pattern. In paper [14], only four patterns (up, bottom, right, and left) are identified by the DC difference and the AC depending on whether they exceed the threshold (on/off). However, the eyeballs angle is not mentioned.
2. In the proposal method, multiple regression analysis and logistic-regression analysis using the DC integral values are performed using the continuous data. Then, discrimination between right and left 30, 60, and 90 degrees and examination of utilization of feature quantity of EOG are carried out.
3. In contrast to paper [14], this proposal method proposes to estimate gaze in a wide space and estimate gaze by using Kinect sensor even if face moves.

### 2.2. Kinect Sensor

In this section, we describe the Kinect sensor used for face position estimation. Several studies have researched the position estimation of faces by using Kinect sensors, but many of these studies have been done in narrow spaces, such as TV screens and PCs [23–25]. In this paper, we used the Kinect sensor to estimate the position of the face in a large space.

**Figure 2:**
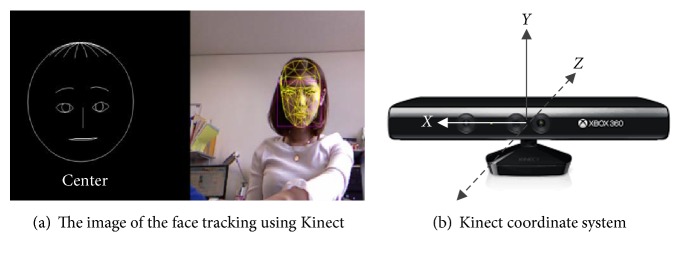
Images of the face tracking system and device.

We chose the Kinect sensor as the RGB-D camera and depth sensors because Kinect sensor is an easy-to-use and low-cost device. The Microsoft Kinect SDK supports face tracking system and its inputs, the color and depth images of the Kinect sensor. The face tracking system was built based on Kinect for Windows SDK and works under C++ programs. An image of the face tracking system is shown in Figure 2(a).

The SDK engine for face tracking analyzes input from the Kinect camera, calculates the face pose and facial expressions, and makes that information available to an application in real time. The face of the target can be projected into 327 feature points, and each part of the face can be reformed as a combination of multiple feature points.

The face tracking SDK uses the Kinect coordinate system to output its 3D tracking results. The origin is located at the camera's optical center, the *z*-axis is pointing towards the user, and the *y*-axis is pointing up and down, as shown in Figure 2(b). The angles are expressed in values ranging from −180 degrees to +180 degrees. The angles of the face are denoted as Rotation *X*, Rotation *Y*, and Rotation *Z*. For example, the angle of the *X*-direction is referred to as Rotation *X*. In this paper, because the face moves sideways, we use Rotation *X*, which outputs *x*-axis data for the face angle.

In order to acquire data simultaneously using Kinect sensor and EOG device, the Kinect sensor is synchronized with the EOG device. The frequency of Kinect sensor is 30 Hz and the EOG device is 500 Hz, so the data of Kinect is synchronized with EOG signal at 30 Hz.

## 3. Method

We carried out experiments with our proposed EOG system to study calculation methods to obtain EOG elements having a strong correlation to the change of eyeball movements. The experiments are carried out by two types: (1) move the eyes only and (2) move the eyes and face.

### 3.1. Extraction Method of Feature Values

#### 3.1.1. EOG Signals

In the experiments using our EOG system, the feature values were (1) AC, (2) DC, (3) DC difference value, and (4) DC integral value.

**Figure 3:**
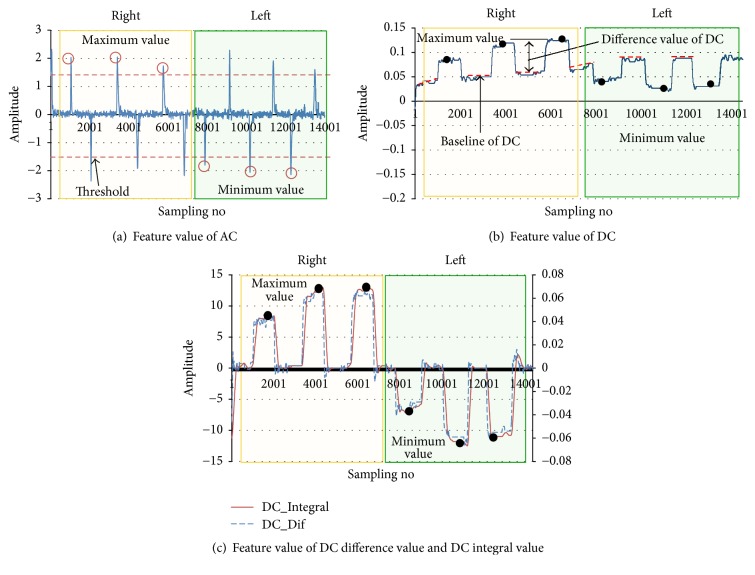
Four types of feature values of EOG signals that were recorded in this study. In (a), AC is the EOG signal recorded in CH3-CH4. In (b) and (c), DC is the EOG signal recorded in CH1-CH2.

*(1) Feature Value of AC*. The feature value of AC is assumed to be the maximum value when AC is more than the threshold in the right direction, and it is the minimum value when AC is less than the threshold in the left direction (Figure 3(a)).

*(2) Feature Values of DC and DC Difference*. The feature value of DC is the maximum value and the minimum value (Figure 3(b)); however, it is necessary to take the difference (DC difference) between the baseline DC value and the DC value because drift occurs in the DC signals. The changing baseline drift makes it difficult to estimate the EOG signals. The baseline (DC_base_) is shown by the dashed line in Figure 3(b).

The DC difference value (DC_dif_) is expressed by (1) where *i* is number of EOG data. When DC_dif_ exceeds a certain threshold value, DC_max_^*R*^ is the maximum values at the time of EOG activity looking to the right direction and DC_max_^*L*^ is taken as the minimum value at the time of EOG activity looking to the left direction. DC_max_^*R*^ and DC_max_^*L*^ are expressed as (2) and (3).

(1) $$\begin{array}{c} {DC}_{dif}\left(\;i\;\right)=DC\left(\;i\;\right)-{DC}_{base}. \end{array}$$

DC_base_ is the baseline DC value, if DC_dif_(*i*) exceeds a certain threshold.

(2) $$\begin{array}{c} {DC}_{max}^{R}=\max{DC}_{dif}\left(\;i\;\right), \end{array}$$

where max⁡DC_dif_(*i*) is the maximum value of DC_dif_(*i*) at the time of EOG activity looking to the right direction.

(3) $$\begin{array}{c} {DC}_{max}^{L}=\min D_{dif}\left(\;i\;\right), \end{array}$$

where min⁡DC_dif_(*i*) is the minimum value of DC_dif_(*i*) at the time of EOG activity looking to the left direction (*i*: 1,2, 3,…, number of EOG data).

*(3) Feature Value of DC Integral Value*. DC integral value (DC_int_) is the linear weighted sum of the DC difference value (DC_dif_) with the baseline subtracted. By taking the maximum/minimum of DC integral value (*X*_max_^*R*^, *X*_max_^*L*^), we can obtain the stable eyes feature value. The DC integral value (DC_int_) is expressed by (4) where *i* is number of EOG data. When DC_int_ exceeds a certain threshold value, *X*_max_^*R*^ is the maximum values at the time of EOG activity looking to the right direction and *X*_max_^*L*^ is taken as the minimum value at the time of EOG activity looking to the left direction. *X*_max_^*R*^ and *X*_max_^*L*^ are expressed as (5) and (6). The dashed line is the value of DC different value and the solid line is the DC integral value (Figure 3(c)).

(4) $$\begin{array}{c} {DC}_{int}\left(\;i\;\right)=\overset{N}{\underset{i=1}{\sum }}{DC}_{dif}\left(\;i\;\right),\;N=200 \end{array}$$

(*i*: 1,2, 3,…, number of EOG data, *N*: 1,2, 3,…, 200).

(5) $$\begin{array}{c} X_{max}^{R}=\max{DC}_{int}\left(\;i\;\right), \end{array}$$

where max⁡DC_int_(*i*) is the maximum value of DC_int_(*i*) at the time of EOG activity looking to the right direction.

(6) $$\begin{array}{c} X_{max}^{L}=\min{DC}_{int}\left(\;i\;\right), \end{array}$$

where min⁡DC_int_(*i*) is the minimum value of DC_int_(*i*) at the time of EOG activity looking to the left direction.

#### 3.1.2. Kinect Sensor

**Figure 4:**
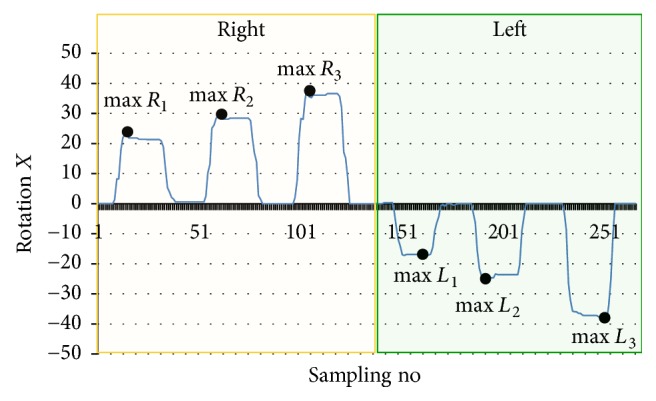
Feature values of data obtained from RGB-D sensor (Rotation *X*).

The feature value of RGB-D data obtained from Kinect sensor was set to the maximum values of Rotation *X* for 30, 60, and 90 degrees and the minimum values of Rotation *X* for −30, −60, and −90 degrees (Figure 4). For example, the maximum values in the right direction are max⁡*R*_1_, max⁡*R*_2_, and max⁡*R*_3_ and the minimum values in the left direction are min⁡*R*_1_, min⁡*R*_2_, and min⁡*R*_3_.

#### 3.1.3. The Synchronization Algorithm between EOG Device and Kinect Sensor

We introduce the synchronization algorithm between EOG device and Kinect sensor. The steps of synchronization algorithm between EOG device and Kinect sensor are as follows.

**Step 1 .**

The EOG element (DC difference, DC integral, or AC) exceeds the reference threshold value.

**Step 2 .**

Our system gets the maximum value (or minimum value) of the EOG element at the time of EOG element being active.

**Step 3 .**

The maximum values (or minimum value) of Rotation *X* from Kinect sensor before 15 data pieces (0.5 seconds) and after 15 data pieces (0.5 seconds) at the time of Step 2 are synchronized with the EOG element maximum value (or minimum value).

Also, when the EOG element falls below the reference threshold for discrimination, gaze information uses the value of the Kinect sensor only. In this algorithm, we do not synchronize when the values of EOG and Kinect sensor are changing by 20% from before data.

### 3.2. Data Analysis

In our previous studies [12, 22], we carried out gaze estimation in the range from −60 degrees to 60 degrees. In this paper, we estimated the gaze from −90 degrees to 90 degrees.

To confirm the accuracy of the EOG system, we performed two types of regression analyses. First is a multiple regression analysis as a linear regression analysis, and the second is a logistic-regression analysis as a nonlinear regression analysis.

We mention sharing ratio briefly; the sharing ratio is a parameter for evaluating the role of face movement and eye movement in gaze movement. Therefore, in multiple regression analysis using explanatory variables as Rotation *X* and EOG elements for gaze estimation, it means estimating gaze considering sharing ratio.

DC is known to have a linear relation [26–28] to the eyeball angle, so we performed a multiple regression analysis with the explanatory variables AC, DC, DC integral value (DC_Int), and DC difference (DC_Dif). Experiments in most previous studies were carried out in a small space, such as a desktop PC [12]. We assumed that DC elements might not have linear shape characteristics in a large space, as in the current experiment. Therefore, we performed a nonlinear logistic-regression analysis in which the explanatory variables were the same as in the multiple regression analysis. We computed the predicted gaze degree by two types of regression analyses for each subject. We compared the explanatory variables to find the most suitable variable for using EOG in a large space.

## 4. Experiment

### 4.1. Experimental Environment

**Figure 5:**
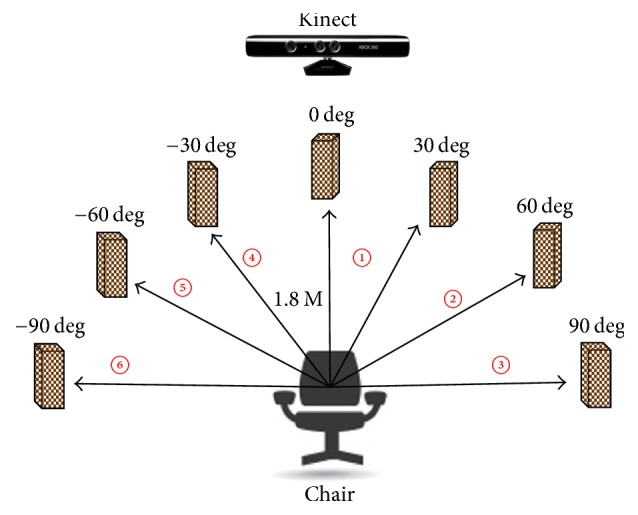
Experimental environment using EOG system and RGB-D sensor (seven boxes and RGB-D sensor).

The experiments were designed to confirm the effectiveness of the proposed system. The experimental condition is shown in Figure 5. We placed seven targets (the targets were the boxes) at 0, 30, 60, 90, −30, −60, and −90 degrees, and an RGB-D sensor (Kinect) was placed at 0 degrees. The subject sat on a chair located 1.8 m away from the targets.

### 4.2. Subjects

We collected data from five healthy subjects (five males) and one patient who participated in this study. The patient is muscular dystrophy. The age of subjects in the experiment is between 22 and 24 years old.

### 4.3. Procedures

We conducted two types of experiments. The first experiment was conducted under the condition that the subject watched a target only with their eyes and without moving his face. The second experiment was conducted under the condition that the subject freely watched an object by using their face and eyes. These two types of experiments as above were conducted on each of the five subjects which was repeated 10 times. For muscular dystrophy patients, these experiments were limited to 3 times, taking into account the patient's burden. We asked the subjects to look at these targets in the order of 0, −30, 0, −60, 0, −90, 0, 30, 0, 60, 0, and 90 degrees, shown as the numbers from 1 to 6 in Figure 5. The time to keep looking at each target was 1 second.

## 5. Experimental Results

In this section, we describe the gaze estimation results of −30 degrees, −60 degrees, −90 degrees, 30 degrees, 60 degrees, and 90 degrees. In the experiments, the proposed analysis method is the major analysis of the target angle of the subjects' views in order to clarify the correlation between the target angle and the error.

To assess the usability of the proposed EOG system, we evaluated the following two factors: target detection accuracy using a correlation coefficient and the error rate throughout the task.

### 5.1. Eye Movement Only

#### 5.1.1. In Case of Healthy Subjects

The results of calculating the respective correlation coefficient *R*^2^ for eye movement only are shown in Table 2. All analysis results with *R*^2^ > 0.835 show a correlation. As shown, DC integral value is highest.

**Figure 6:**
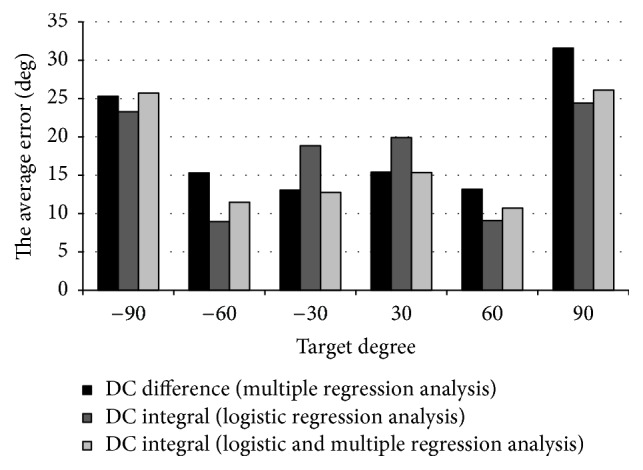
Average errors of eyes movement only: the horizontal axis represents the target angle and the vertical axis represents the average error.

Based on the results of the multiple regression analysis, we conducted a logistic-regression analysis with DC integral value. In addition, we calculated the average errors between the predicted values and the true values obtained by the multiple regression analysis and the logistic-regression analysis. The average errors of all data at the same target angle are shown as a bar graph in Figure 6.

Figure 6 shows that 60 degrees and −60 degrees have small average errors for each angle and each type of analysis on average. The gaze estimation by the nonlinear analysis is better for angles larger than 60 degrees or less than −60 degrees. In the results of all data from −90 degrees to 90 degrees, the error rate of the multiple regression analysis is 19.0 and the error rate of the logistic-regression analysis is 17.4.

By using the best experimental results, the success rate in each target angle of −90 degrees is 24%, −60 degrees is 71%, −30 degrees is 66%, +30 degrees is 50%, +60 degrees is 83%, and +90 degrees is 5%. The eyeball angles of 60 degrees and −60 degrees are the most easy to judge by the EOG, and consequently the success rate is 77% without considering the individual differences of the five subjects. For the average error at ±60 degrees, only one subject is over 15, and it is considered that the success rate is decreasing due to the influence. At 90 degrees and −90 degrees, the judgment is difficult because the individual differences are wide and the value of the EOG tends to be saturated. At about 30 degrees and −30 degrees, two of the five subjects show 80% success rates. One of the causes for a low success rate is the influence of the individual differences.

**Figure 7:**
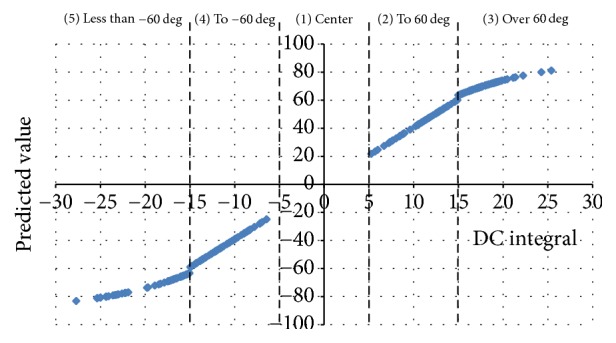
Relation between the predicted values and DC integral value.

Figure 7 shows that the existence of a linear relation of the DC value and eyeball angle depends on the eyeball angle. Therefore, we established a boundary line (DC integral value: ±15 V) to separate the linearity and nonlinearity and combined the results of the logistic-regression analysis and the multiple regression analysis (Figures 6 and 7). Based on 60 degrees, our linear and nonlinear analysis methods can be classified as the following patterns as shown in Figure 7: (1) center, (2) center to 60-degree range, (3) 60 degrees and over, (4) center to −60-degree range, and (5)  −60 degrees and less. For our linear and nonlinear analysis methods, it can be said that 5 is the appropriate number of judgment patterns.

#### 5.1.2. In Case of Muscular Dystrophy Patient

We conducted experiments with muscular dystrophy patients under the same experimental environment and experimental contents as healthy subjects. However, taking the burden on the patient into consideration, the number of experiments was set three times.

**Figure 8:**
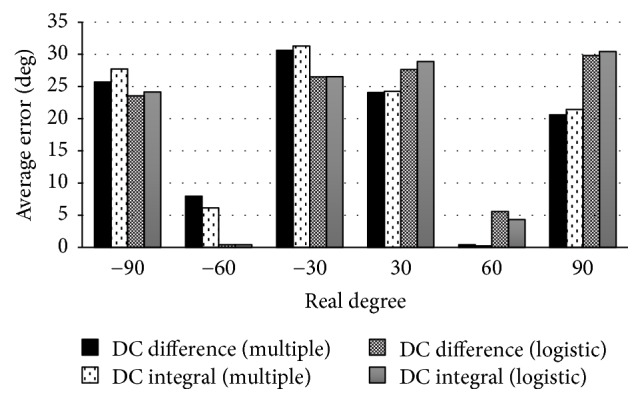
Average errors of eyes movement only in case of the muscular dystrophy patient: the horizontal axis represents the target angle and the vertical axis represents the average error.

The results of calculating the respective correlation coefficient *R*^2^ for eye movement only are as follows: AC value is 0.839, DC difference value is 0.894, and DC integral value is 0.887. Based on the results of the multiple regression analysis, we conducted a logistic-regression analysis with DC difference value and DC integral value. In addition, we calculated the average errors between the predicted values and the true values obtained by the multiple regression analysis and the logistic-regression analysis. The average errors of all data at the same target angle are shown as a bar graph in Figure 8.

The same as in healthy subjects, the average error of ±60 degrees is under 15 which is the smallest average error in each target degree, and ±30 degrees and ±90 degrees have larger average errors in all analysis methods.

### 5.2. Both Eye and Face Movements

In this section, we show the experimental results when the face freely moved.

#### 5.2.1. In Case of Healthy Subjects

We show the multiple regression analysis results in Table 3. AC, DC difference, DC integral value, and Rotation *X* are the explanatory variables. We performed a multiple regression analysis with two explanatory variables, DC integral value and Rotation *X*. All results with *R*^2^ > 0.804 show a correlation for all analyses. In the case of one explanatory variable, the DC integral value is the highest value in 4 out of 5 people, but the result in the case where the explanatory variable is two of Rotation *X* and DC integral value is the best result in all the analysis results.

**Figure 9:**
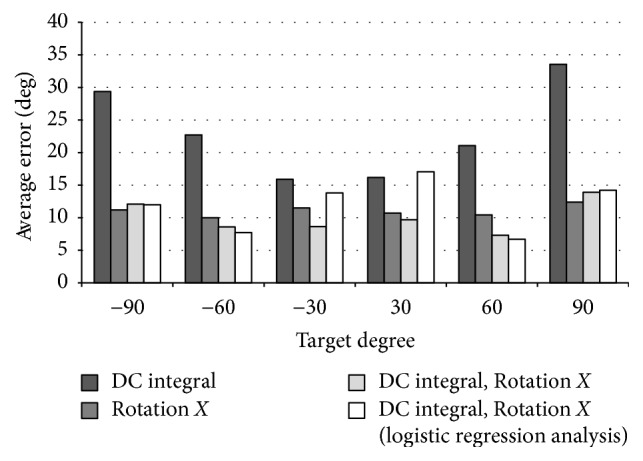
Average errors of both eye and face movements: the horizontal axis represents the target angle and the vertical axis represents the average error.

We calculated the average errors between the predicted values and the true values obtained by the multiple regression analysis and the logistic-regression analysis (Figure 9).

The best average error result of 60 degrees is the result of logistic-regression analysis with Rotation *X* and DC integral value as explanatory variable, and the average error of ±60 degrees is both under 15.

By using the best experimental results to compare this experiment (both eye and head movements) with the experiment of only eye movement, the success rate of this experiment (both eye and head movements) is 81% on average. This success rate is 39% better than the experiment with eye movement only. The lowest value is 67% in the cases of +90 degrees; two of the five subjects had an average error of 15 or more. The reason of lowing the average error is considered to be that there are variations in Rotation *X* values. Furthermore, in case of ±60 degrees, it was more than 90%. All of these results are better than those in the experiment of eye movement only.

#### 5.2.2. In Case of the Face Inclined

The subjects in the experiments were healthy subjects, but the aim is to use this system for patients with brain disease and patients with disabilities such as muscular dystrophy patients. Patients with brain disease may not be able to keep their faces straight, so another experiment was performed with the face tilted to assume a possible condition of a patient. The subject of this experiment is Subject B, who tilted his face about 40 degrees to the right.

**Figure 10:**
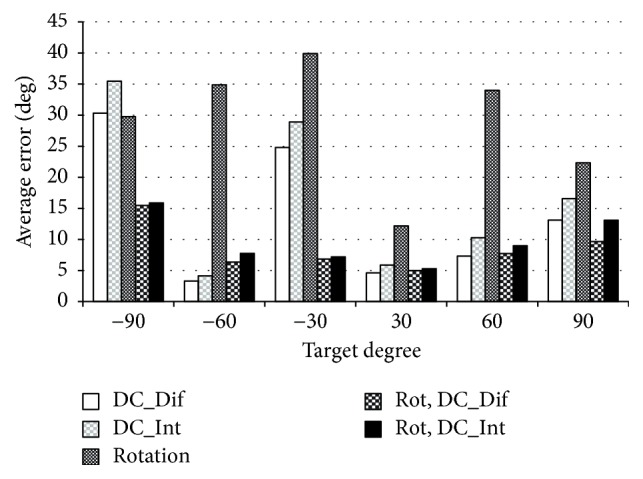
Average errors of face inclination: the horizontal axis represents the target angle and the vertical axis represents the average error.

Table 4 shows the multiple regression analysis results of the correlation coefficients in the case of the face inclined. Since the multiple regression analysis results were better than the logistic-regression analysis results, the mean error was calculated for the multiple regression analysis. These results are shown in Figure 10.

When the face is tilted, the correlation coefficient between Rotation *X* obtained from Kinect and the true value (the targets angle) is lower as compared with the result of the not inclined face which is mentioned in Section 5.2.1. The result of the multiple regression analysis with DC integral value and Rotation *X* as explanatory variables is a good result. This result is similar to the result of the face without an incline in the basic experiment.

In the mean error, multiple regression analysis with Rotation *X* and DC difference as the two inputs had the smallest average error overall. The average error is less than 10 for all degrees of the targets. Because of the inclination of the face, the correlation between Rotation *X* and the true value is low, and the average error is the largest except for −90 degrees. Therefore, when using this system for patients with inclined faces, using the Kinect sensor with the EOG system is better than using the Kinect sensor alone.

#### 5.2.3. In Case of Muscular Dystrophy Patient

**Figure 11:**
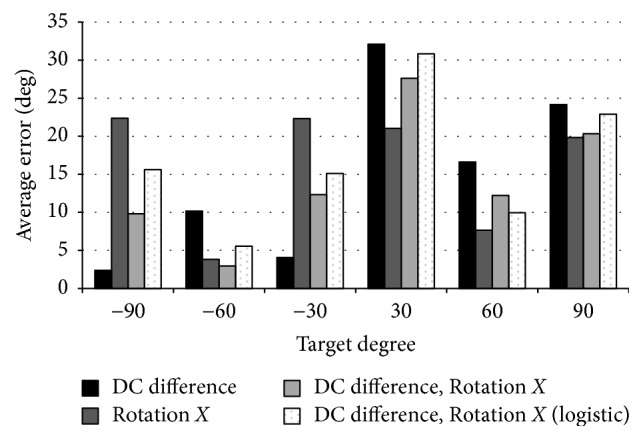
Average errors of both eye and face movements in case of the muscular dystrophy patient: the horizontal axis represents the target angle and the vertical axis represents the average error.

The results of calculating the respective correlation coefficient *R*^2^ for eye movement only are as Table 5 and the average error is shown in Figure 11.

The result of multiple regression analysis with Rotation *X* and DC difference value as explanatory variable was the higher result.

In each of the left and right directions, the average error of 60 degrees is the smallest. The average error of ±60 degrees is under 15, which is the same for the results of healthy subjects. In the right direction (+30, +60, and +90 degrees), the error is the smallest error of Rotation *X*. On the other hand, regularity was not observed in the left direction (−30, −60, and −90 degrees). This is because it is thought that the error of Rotation *X* increases because the subject can move the face a little to the left when the subject moves the face. However, since the error of Rotation *X* is small if the angle is 60 degrees to the left and right, this subject can estimate the gaze with high performance by setting the viewing target at ±60 degrees when moving the face.

## 6. Discussion

**Figure 12:**
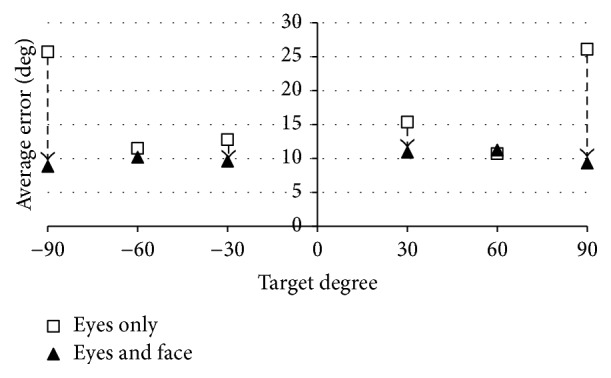
Average errors of two types of experiments: eye movement only and both eye and face movements.

In this section, we discuss the effectiveness of moving the face versus not moving the face.  Figure 12 shows the average errors between the estimated values and the true values of the regression analysis results in the experiments of only eye movement and both eye and face moving. The results in Figure 12 used the data of all five subjects.

The average error of the face moving condition is smaller, and even the inclined face (average error: 9) is a better result than that for subjects using their eyes only to look at the target objects. A statistically significant difference using the *t*-test is observed between the experiment of using only eye movement and that of moving the face (*p* < 0.01). From the above, we can say that the average error is reduced by moving the eyes and the face. Gaze estimation when moving both the face and the eyes is thus possible with stable accuracy.

When trying to see the object by only eye movement, the error becomes large except for ±60 degrees. For 30 degrees, as mentioned in Section 5, the average error is large because of the individual differences. The viewing angle at which motion vision works effectively is about 60 degrees horizontally [29, 30] and looking at an object without moving the face means capturing an object only with peripheral vision. Therefore, we think that 90 degrees exceeds the limit of the angle for looking at an object with only the eyes.

In the case of this patient, when comparing the case where the face was moved and the case where only the eye was moved, the average error was improved at ±90 and ±30 degrees: −90 degrees was 65%, −30 degrees was 25%, 30 degrees is 29%, and 90 degrees is 64%. From these facts, it is considered that gaze estimation is possible with stable precision by moving the face even for patients who can move their faces a little.

Furthermore, using regression analysis considering EOG and Kinect information (sharing ratio), gaze estimation is superior to only EOG, Kinect only. In addition, we compared our proposal method using regression analysis with the nonlinear model. We used the adaptive neuro fuzzy inference system [31]. The correlation coefficient when eye and face were moved freely was *R*^2^ = 0.942. This result was lower than the correlation coefficient of the multiple regression analysis of this paper. Therefore, our proposal method does not need to use a nonlinear model.

## 7. Conclusions

In this paper, we conducted experiments to examine EOG elements that have a strong correlation with eye movement changes. Furthermore, we examined the accuracy of gaze estimation by using the face and the eyes. From the experiments, we established the following three points in this study.

1. With only eyes movement, the position of ±60 degrees is the most accurate. If we set the objects to see in these two places, we can recognize it without setting the range for each individual. DC integral value is the most effective EOG signal for gaze estimates using only the eyes. When a bedridden person whose face does not move can be used, recognition at this angle is possible.
2. In the case of including face motion, estimation is possible since the average error is less than 15 in 6 patterns of −90 to 90 degrees' gaze estimation. We reported [22] that the success rate is 50% when only EOG is used and 65% when only Kinect is used, so the estimation is difficult only with Kinect, EOG alone.
3. The tendency of (1) and (2) is the same in patients with muscular dystrophy.

When gaze estimation is performed in a large space, we know that the gaze estimation of 60 degrees in the left and right directions is the most stable. Therefore, by arranging the objects at ±60 degrees, 5 patterns can be input when only eye movement is used, and 7 patterns can be input when using both face and eye movements for healthy subjects. In the case of the muscular dystrophy patient this time, it is possible to input 3 patterns only by movement of eyes and 3 patterns when considering the movement of the face.

We found that it is difficult to estimate the gaze more accurately with only eye movement. We thought that moving the face supplemented the missing movements of the eyes, and so moving both the face and the eyes improves the success rate.

In addition, because looking at objects by using only the eyes leads to overuse of the eyes, we think that using both face and eye movements is desirable for patients who can move their faces in a natural state. Also, as can be seen from the results, the individual differences were large in past experiments [22]. However, because the average error could be reduced to 10 degrees or less without considering individual differences, this system could possibly unify gaze estimations in the future. Furthermore, it was found that good discrimination accuracy can be obtained in this muscular dystrophy patient if it is ±60 degrees. By setting the viewing target at a location of 60 degrees, it becomes possible to develop an application that allows the patient himself/herself to turn on and off the switch by looking at the place by the patient.

For these reasons, development of a system that can be easily used without making precise settings for each individual patient is a future subject. Then, the gaze estimation system of this study could be used as a communication tool for patients with brain disease.

## Figures and Tables

**Table 1:** The electrodes positions of our method and the conventional method.

	Positions of the electrodes
Conventional method	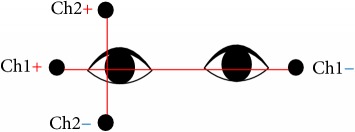

Proposed cross-channel method	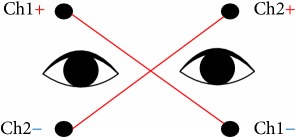

**Table 2:** Correlation coefficients of multiple regression analysis for eye movement only.

	AC	DC difference	DC integral
Subject A	0.835	0.790	0.925
Subject B	0.844	0.908	0.922
Subject C	0.845	0.845	0.906
Subject D	0.841	0.911	0.914
Subject E	0.823	0.927	0.930

**Table 3:** Correlation coefficients of multiple regression analysis of face and eye movements.

	AC	DC difference	DC integral	Rotation *X*	DC integral, Rotation *X*
Subject A	0.851	0.910	0.971	0.965	0.975
Subject B	0.848	0.957	0.973	0.984	0.988
Subject C	0.864	0.944	0.944	0.933	0.959
Subject D	0.804	0.864	0.835	0.942	0.964
Subject E	0.845	0.931	0.961	0.942	0.974

**Table 4:** Correlation coefficients of the face inclined experiment.

	DC difference	DC integral	Rotation *X*	DC integral, Rotation *X*	DC difference, Rotation *X*
Correlation coefficient	0.918	0.887	0.740	**0.968**	0.963

**Table 5:** Correlation coefficients of multiple regression analysis of face and eye movements (muscular dystrophy patient).

AC	DC difference	DC integral	Rotation *X*	DC difference, Rotation *X*
0.874	0.912	0.865	0.915	0.935
